# Supplementary material for: Dopaminergic co-transmission with sonic hedgehog inhibits abnormal involuntary movements in models of Parkinson’s disease and L-Dopa induced dyskinesia
Source: Commun Biol. 2021 Sep 22;4:1071. doi: 10.1038/s42003-021-02567-3 (PMC8458306; doi:10.1038/s42003-021-02567-3)
Supplement: Supplementary file 2 — Supplementary Information [file 42003_2021_2567_MOESM2_ESM.pdf]

Supplementary Information for

"Dopaminergic Co-transmission with Sonic Hedgehog Inhibits Abnormal Involuntary Movements in Models of Parkinson's Disease and L-Dopa Induced Dyskinesia"

Lauren Malave, Dustin R. Zuelke, Santiago Uribe-Cano, Lev Starikov, Heike Rebholz, Eitan Friedman, Chuan Qin, Qin Li, Erwan Bezard, and Andreas H. Kottmann.

Supplementary Figures:

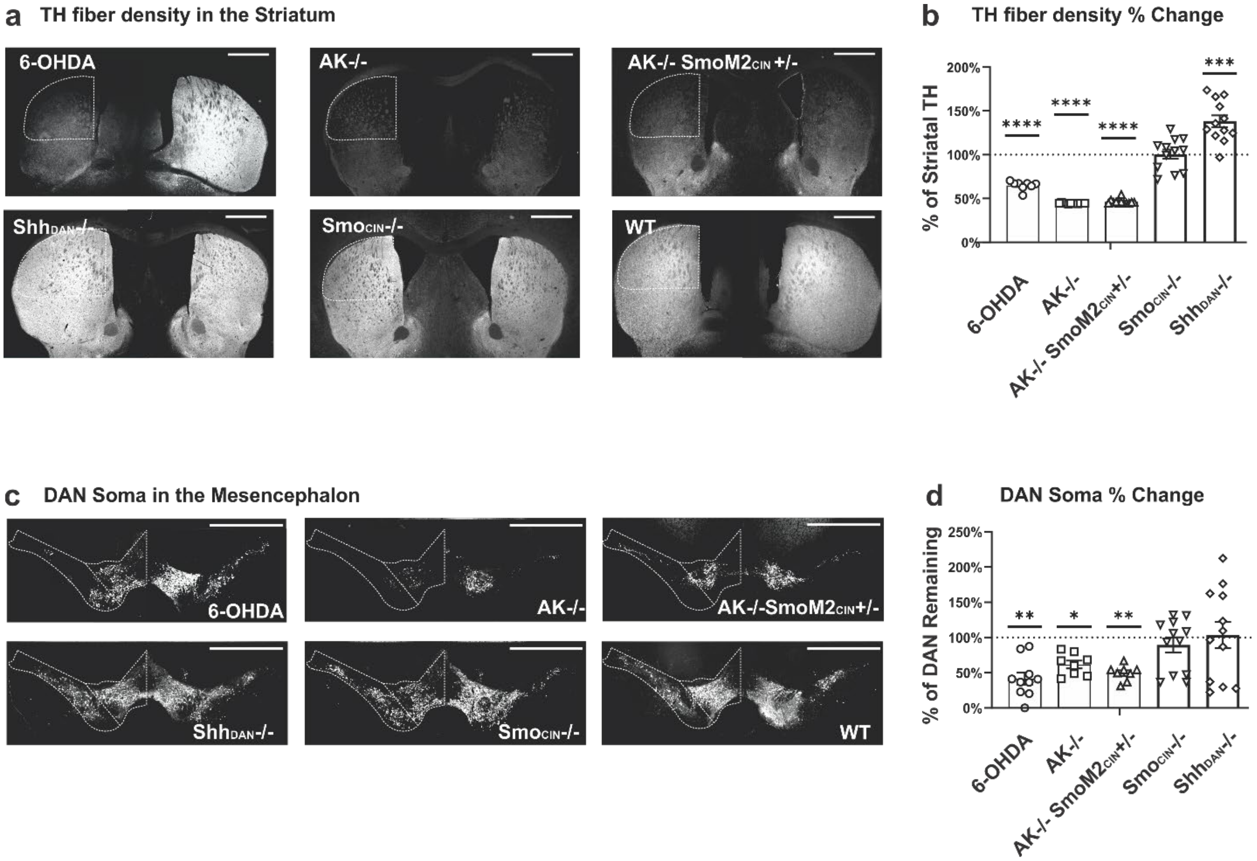

Supplementary Figure 1: TH fiber density or numbers of midbrain DAN do not predict LID formation and expression.

a Tyrosine hydroxylase (TH) fiber density in the dorsolateral striatum (DLS, dotted quarter circle) was visualized as a proxy for the integrity of dopaminergic projections across the mouse models utilized in this study (Size bar: 500 µm). **b** For quantification of Striatal TH fiber density, TH staining intensity was normalized to background signal and average TH intensity in the DLS per animal was reported. Results are reported as percent difference between experimental animals and average TH intensity of control animals (n = 8–12, 3-5 planes each; unpaired two-tailed student's t test **** P<0.0001 6-OHDA lesioned hemisphere vs. 6-OHDA unlesioned hemisphere, *$AK^{-/-}$* vs *WT*, *$AK^{-/-}$ $SmoM2_{CIN}{}^{+/-}$* vs *W*T, *$Smo_{CIN}{}^{-/-}$* vs *$Smo_{CIN}{}^{+/-}$*; *** P<0.001 *$Shh_{DAN}{}^{-/-}$* vs *$Shh_{DAN}{}^{+/-}$***.** All bar graphs are plotted as mean +/- SEM. **c** DAN cell bodies were identified as TH positive cell soma on coronal sections of the mesencephalon in all mouse models used in this study (Size bar: 500 µm). **d** For quantification of Midbrain DAN Soma, TH background signal was set as a threshold and all soma in the VTA and SNpc with TH signal above background were counted. Results are reported as percent difference in number of TH+ soma between experimental animals and control animals (n = 8–12, 3-5 planes each; unpaired two-tailed student's t test ** P<0.01 6-OHDA lesioned hemisphere vs. 6-OHDA unlesioned hemisphere, *$AK^{-/-}$ $SmoM2_{CIN}{}^{+/-}$* vs *WT*, * P<0.05 *$AK^{-/-}$* vs ***WT*.** All bar graphs are plotted as mean +/- SEM.

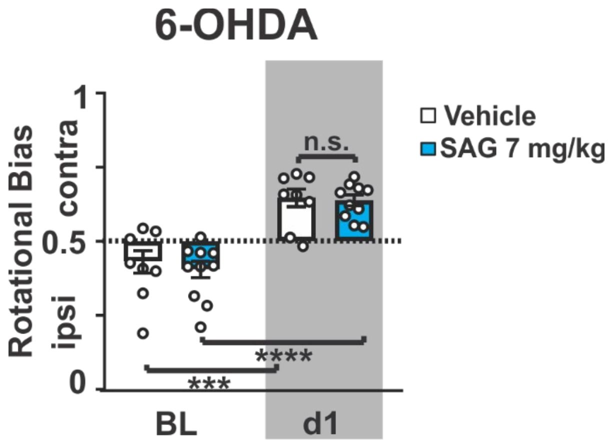

Supplementary Figure 2: Functional validation of the unilateral 6-OHDA lesion model.

Quantification of rotational bias as a ratio of contra- to ipsilateral turns. Dotted line signifies the absence of turning bias. Mice with 6-OHDA lesions (baseline: BL, white bar) turned ipsilateral to the lesion, indicative of hypodopaminergia in the lesioned hemisphere. Upon L-Dopa (5 mg/kg) dosing, mice turned contralateral towards the lesion, suggesting dopamine hypersensitivity had formed in the lesioned hemisphere (d1, white bar). Co-injection of L-Dopa with Smo agonist SAG (7 mg/kg, blue bars) did not alter turning bias caused by L-Dopa alone. (n = 8–9; RM two-way ANOVA time effect: $F_{(2,50)} = 20.19$, P<0.0001. Post hoc Bonferroni's test: *** P<0.001 BL vs. day 1). Bar graphs are plotted as mean +/- SEM.

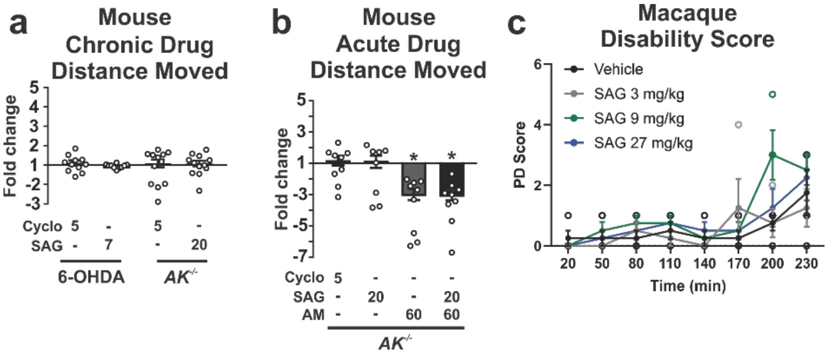

Supplementary Figure 3: SAG treatment does not curtail the anti-parkinsonian benefit of L-Dopa

a Fold change of distance moved in an open field arena following daily co-injection of L-Dopa and either Cyclopamine (5 mg/kg) or SAG (7 mg/kg for 6-OHDA mice or 20 mg/kg in *AK*$^{-/-}$ mice) in 6-OHDA (day 14 of treatment; n = 8) and *AK*$^{-/-}$ (day 26 of treatment; n = 12) mice. Results are reported as fold change over vehicle-treated controls. Cyclopamine or SAG co-injection with L-Dopa in 6-OHDA treated or *AK*$^{-/-}$ mice did not alter locomotion activity compared to L-Dopa + vehicle controls. **b** Fold change of distance moved on day 20 of daily L-Dopa injections *AK*$^{-/-}$ mice that received an acute dose of either Cyclopamine (5 mg/kg; n = 9), SAG (20 mg/kg; n = 8), Amantadine (AM, 60 mg/kg; n=9), or a combination of AM (60 mg/kg) and SAG (20 mg/kg; n = 8). AM significantly reduced the anti-akinetic benefit of L-Dopa but SAG did not (unpaired two-tailed student's t test: * $P<0.05$ for treatment vs. vehicle. n.s. indicates $P>0.05$). **c** Parkinsonian disability score of Parkinsonian Macaques across a four-hour time course after receiving L-Dopa together with either vehicle or SAG (3, 9, and 27 mg/kg; n = 4 per condition). Scoring involved evaluating a range of movements including bradykinesia, postural abnormality, and tremor, yielding a maximum global parkinsonian disability score of 10 (Methods). There was no effect of SAG on the Parkinsonian disability score signifying that SAG did not diminish the akinetic benefit of L-Dopa treatment. All graphs are plotted as mean +/- SEM.

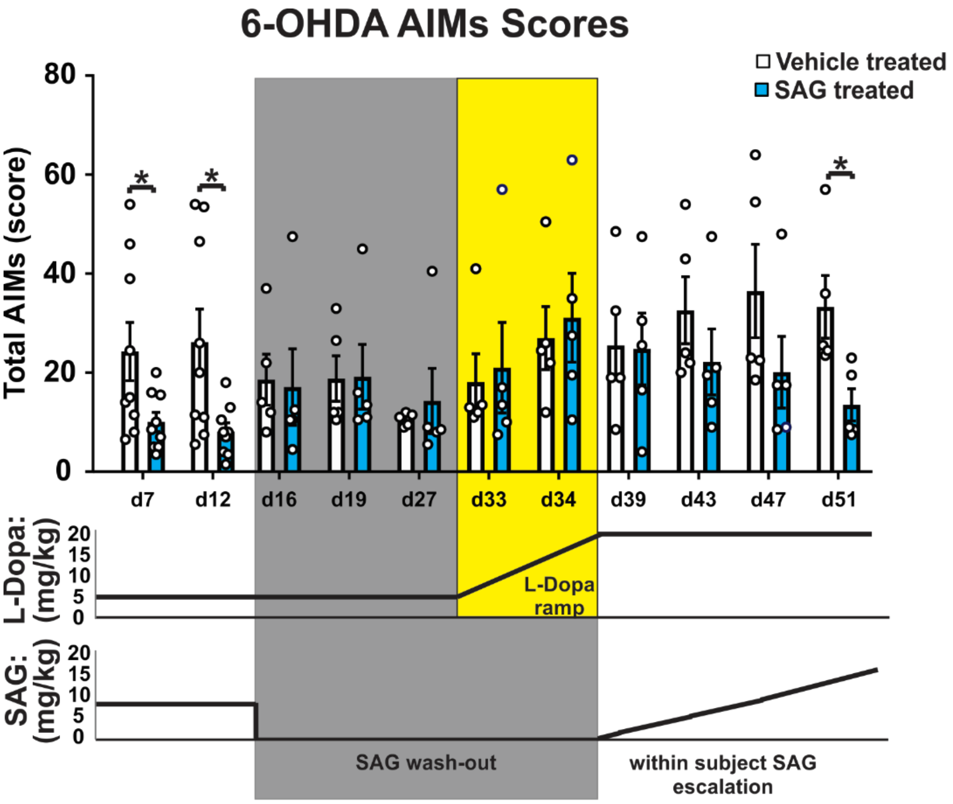

Supplementary Figure 4: AIMs can be repeatedly attenuated or reinstated through sequential dosing of L-Dopa with or without SAG, respectively.

6-OHDA animals co-injected daily with L-Dopa and Smo agonist SAG (5 mg/kg) for seven days showed significantly attenuated LID compared to L-Dopa only injected controls. Additional days of L-Dopa and SAG co-administration did not further reduce LID (d12). Terminating SAG dosing on day 16 while continuing L-Dopa treatment resulted in an immediate reappearance of LID to levels seen in controls. Severity of reinstated LID remained sensitive to L-Dopa concentration such that a gradual increase in L-Dopa dosing led to greater AIM scores with no difference in kinetics or absolute intensity compared to controls (d33–d34). Reinstated LID could be attenuated again in a dose-dependent manner through within-subject escalation of SAG on days 39–51. During this within-subject SAG escalation, three-day SAG washout periods during which only L-Dopa was administered were included between days of increasing SAG dose administration (n = 9 for d 7, 12; n = 5 for d 16–51; Paired two-tailed student's t test: * $P<0.05$ treatment vs. vehicle. n.s. indicates $P > 0.05$). Bar graph is plotted as mean +/- SEM.

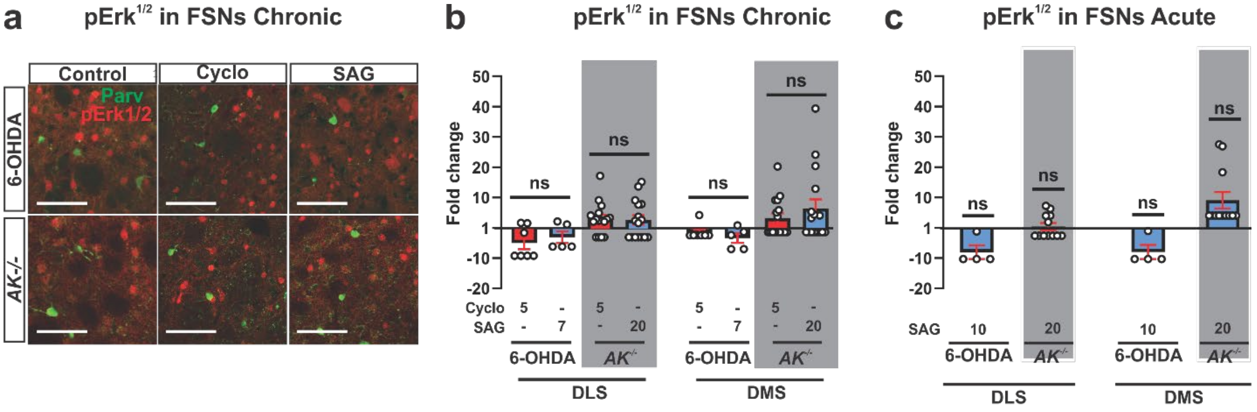

Supplementary Figure 5: Smo activity does not modulate the MAP Kinase pathway in FSN

a Representative images from DLS of 6-OHDA or *AK*$^{-/-}$ mice revealing no evidence for co-localization of the cytohistological LID marker pErk$^{1/2}$ (red) with the FSN marker Parvalbumin (Parv, green) after repeated co-administration of L-Dopa (5 mg/kg for 6-OHDA or 25 mg/kg for *AK*$^{-/-}$ animals) with vehicle (control), cyclo (5 mg/kg), or SAG (7 mg/kg for 6-OHDA mice or 20 mg/kg for *AK*$^{-/-}$ mice). Images were taken from DLS of animals whose AIMs were quantified in panels **b** and **c** of Figure 1. (Scale bar = 50 μm). **b** Quantification of the prevalence of pErk$^{1/2}$-positive FSNs shown in (Figure 1**g**) expressed as fold change over vehicle in the DLS and DMS. Cyclopamine or SAG treatment did not alter pErk$^{1/2}$ prevalence in the DLS or DMS (n= 7-15 per condition; 3 sections each; ~36 CIN per section, post-mortem analysis of animals quantified in Figure 1 **b** and **c**; unpaired two-tailed student's t test: * P<0.05 treatment vs. vehicle. n.s. indicates P>0.05). **c** Quantification of the prevalence of pErk$^{1/2}$-positive CIN in L-Dopa treated 6-OHDA (5 mg/kg L-Dopa for 14 days) or *AK*$^{-/-}$ (25 mg/kg L-Dopa for 20 days) animals given a single dose SAG (10 mg/kg for 6-OHDA animals, 20 mg/kg for *AK*$^{-/-}$ animals). Smo agonist treatment did not significantly alter the prevalence of pErk$^{1/2}$-positive FSN in the DLS or DMS (n= 5-13 per condition; 3 sections each; ~36 CIN per section, post-mortem analysis of animals whose AIMs were quantified in Figure 1 **d** and **e**; unpaired two-tailed student's t test: * P<0.01, *** P<0.001, for treatment vs. vehicle. n.s. indicates P>0.05). All bar graphs are plotted as mean +/- SEM.

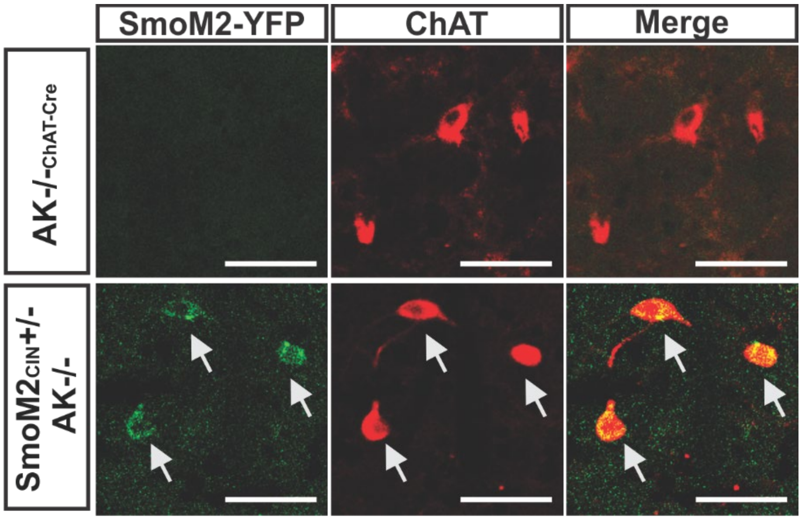

Supplementary Figure 6: Conditional expression of SmoM2 in CIN of *AK*$^{-/-}$ mice.

Images of the DLS showing eYFP-tagged SmoM2 expression selectively in CIN of *SmoM2*$_{CIN}^{+/-}$*AK*$^{-/-}$ (arrows) but not in *AK*$^{-/-}$ $_{ChAT\text{-}Cre}$ littermate controls. CIN identified through co-labeling with ChAT. (Scale bar = 50 µm).

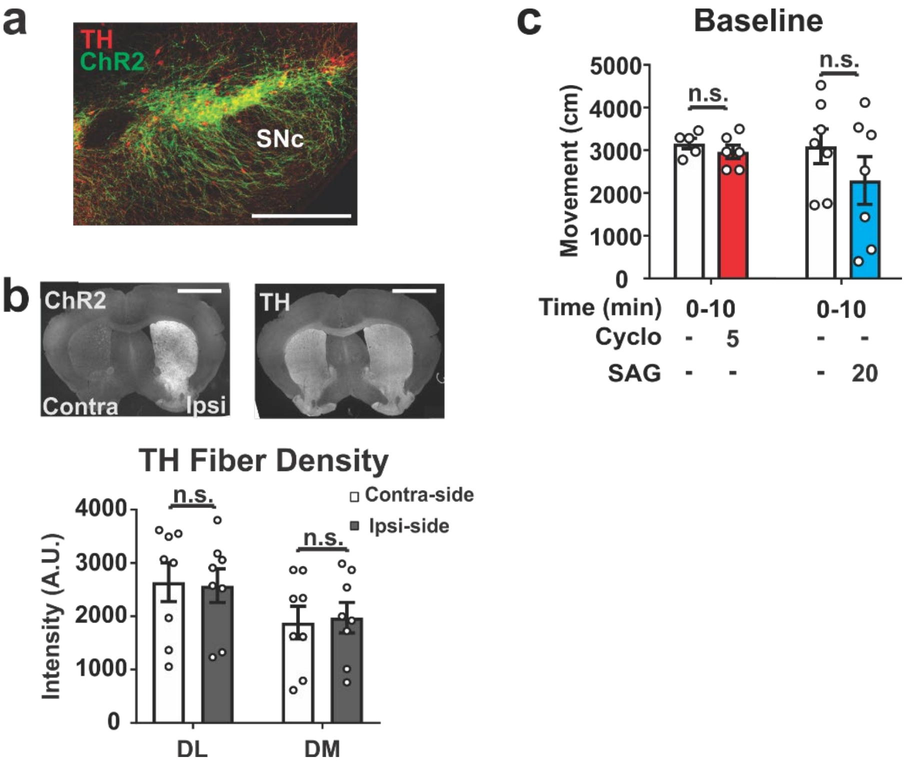

Supplementary Figure 7: Daily repeated, long-term optogenetic stimulation of DAN does not reduce dopaminergic fiber density in the striatum.

a Co-localization of the channelrhodopsin (ChR2):: eYFP (green) fusion protein and TH (red) in the SNpc of Dat-Cre$^{+/-}$ animals (scale bar = 500 µm). **b** Representative images and quantification of TH fiber density after daily, hour long optical burst stimulation of DAN for 10 days. Comparison is made between the hemisphere ipsilateral to ChR2 expression and the contralateral striatum (n = 8; n.s. indicates P > 0.05; scale bar = 1 mm). **c** Neither Cyclopamine (5 mg/kg) nor SAG 20 (mg/kg) injection altered total locomotion displacement in the Open Field paradigm. All bar graphs are plotted as mean +/- SEM.

Supplementary Tables:

Supplementary Table 1: Summary of dosing regiments and cyto-histochemical analysis for each paradigm.

Paradigm	L-Dopa [mg/kg]	Cyclopamine [mg/kg]	SAG [mg/kg]	Purmorphamine [mg/kg]	$pERK_{CIN}$ levels	$p\text{-}rpS6_{CIN}$ levels
**6-OHDA**	5 or<br>5; 10; 15;20	5	0.8; 2.5; 7	15	yes	n/d
**AK-/-**	25 or<br>5; 10; 30	5	20 or<br>0.8; 2.5; 7; 15	n/d	yes	n/d
**Macaques**	18 - 22	n/d	3; 9; 27	n/d	n/d	n/d
**Shh_{DAN}-/-**	10 or 25	n/d	10 or 20	n/d	yes	yes
**Smo_{CIN}-/-**	25	n/d	n/a	n/d	yes	yes
**$SmoM2_{CIN}$+/-**	25	n/d	n/a	n/d	yes	yes
**Optogenetic DAN activation**	n/a	5	20	n/d	n/d	yes

Supplementary Table 2: Summary of genotypes of experimental and control animals of all recombinant mouse lines and their ages at time of analysis.

Paradigm	Genotype experimental	Genotype control	Age dosing	Age LID analysis	Age Erk analysis	Age TH fiber/DAN analysis
Shh_{DAN}-/- vs. *Shh_{DAN}+/-*	Shh-nLacZ$^{L/L}$; Dat-Cre	Shh-nLacZ$^{L/+}$; Dat-Cre	2-3	3	3	3; 1 – 18 [1]
Smo_{CIN}-/- vs. *Smo_{CIN}+/-*	Smo$^{L/L}$; ChAT-IRES-Cre	Smo$^{L/+}$; ChAT-IRES-Cre	2-3	3	3	3
$SmoM2_{CIN}$+/-; AK-/- vs. *ChatCre AK-/-*	^{L}STOP^{L}SmoM2-YFP; ChAT-IRES-Cre; Pitx3$^{ak/ak}$	ChAT-IRES-Cre; Pitx3$^{ak/ak}$	2-3	3	3	3

Supplementary Table 3: Strain specific genotyping PCR amplimers.

**Gene**	**Oligo Forward**	**Oligo Reverse**
Shh	GTAAGAGCACATTACCCAGAGAACTG	CCTGTTGTTACTGGATCCCTTCCATC
Generic Cre	TAGCGCCGTAAATCAATCG	AATGCTTCTGTCCGTTTGC
$Smo^{fl/fl}$	ATGGCCGCTGGCCGCCCCGTG	GGCGCTACCGGTGGATGTGG
Smo WT	CCACTGCGAGCCTTTGCGCTAC	CCCATCACCTCCGCGTCGCA
SmoM2 Mutant	CTGACCCTGAAGTTCATCTGC	GTGCGCTCCTGGACGTAG
SmoM2 WT	CGTGATCTGCAACTCCAGTC	GGAGCGGGAGAAATGGATATG
ChAT-Cre	CAGGGTTAGTAGGGGCTGAC	CAAAAGCGCTCTGAAGTTCCT

Supplementary Table 4: Reagents and Resources:

Reagent or Resource	Source	Identifier
**Antibodies**		
Rabbit anti-TH	Millipore	657012
Chicken anti-B Galactosidase	Millipore	AB986
Goat anti-chat	Millipore	AB144P
Goat anti-parv	Swant	PVG-213
Rabbit anti-p-p44/42 MAPK (Erk1/2)	Cell Signaling Technology	9101
Rabbit anti-cfosB	Cell Signaling Technology	2250
Rabbit anti-p-(Ser/Thr)PKA substrate	Cell Signaling Technology	9621
GP anti-vGlut1	Dr. Thomas Jessell from Columbia Laboratory	
GP anti-vGlut2	Millipore	AB2251-1
Rabbit anti-PSD95	Invitrogen	51-6900
Rabbit anti-p-rpS6	Cell Signaling Technology	5364
Mouse anti-NeuN	Chemicon international	MAB377
Alexa Fluor 488- Donkey Anti-Goat IgG	Jackson ImmunoResearch	705-545-147
Alexa Fluor Cy3- Donkey Anti-Goat IgG	Jackson ImmunoResearch	705-165-147
Alexa Fluor Cy3- Donkey Anti-Rabbit IgG	Jackson ImmunoResearch	711-165-152
Alexa Fluor 594- Donkey Anti-Rabbit IgG	Jackson ImmunoResearch	711-585-152
Alexa Fluor Cy5- Donkey Anti-Mouse IgG	Jackson ImmunoResearch	715-175-150
Alexa Fluor Cy5- Donkey Anti-GP IgG	Jackson ImmunoResearch	706-175-148
Alexa Fluor Cy5- Donkey Anti-Chicken IgG	Jackson ImmunoResearch	703-175-155
**Virus Strains**		
AAV5-EF1α-DIO-eYFP	University of North Carolina Vector Core	
**Chemicals, Peptides, and Recombinant Proteins**		
6-Hydroxydopamine hydrobromide	Sigma-Aldrich	H4381
Smoothened Agonist (SAG)	Carbosynth Limited	FS27779
SAG-HCl	Carbosynth Limited	FS76762
Cyclopamine	Carbosynth Limited	FC20718
VECTASHIELD Antifade Mounting Medium	Vector Laboratories	Cat# H-1000, RRID: AB_2336789
Neg-50	Thermo Scientific	6506M
C&B-Metabond	Parkell	S380
Benserazide hydrochloride	Sigma-Aldrich	B7283

L-3,4-Dihydroxyphenylalanine methyl ester hydrochloride	Sigma-Aldrich	D1507
Hydroxypropyl-β-cyclodextrin (HPCD)	Sigma-Aldrich	H107
Amantadine hydrochloride	Sigma-Aldrich	A1260
Ascorbic acid	Sigma-Aldrich	A92902
Bupivacaine (Murcaine)	Sigma-Aldrich	B5274
M4PAM (VU0467154)	StressMarq Biosciences	SIH-184
Purmorphamine	ABCam	Ab120933
**Experimental Models: Organisms/Strains**		
Mouse: WT: C57BL/6J	The Jackson Laboratory	RRID: IMSR_JAX:000664
Mouse: Shh-nLZ$^{C/C}$	The Jackson Laboratory	Gonzalez-Reyes et al., 2012RRID: IMSR_JAX:000664
Mouse: Shh-nLZ$^{C/C}$/Dat-Cre Slc6a3tm1(cre)Xz/JMouse: Shh-nLZ$^{C/C}$	The Jackson Laboratory	Stock No: 020080
Mouse: myrGFP; Shh-cre	The Jackson Laboratory	
Mouse: Pitx3ak/ak	Dr. Un Jung Kang from Columbia University	
Non-human primates: *Macaca Fascularis*	Xierxin, Beijing, PR of China	
**Software and Algorithms**		
ImageJ	NIH	https://imagej.nih.gov/ij/; RRID: SCR_003070
EthoVision 10 XT	Noldus	http://www.noldus.com/animal-behavior-research/ products/ethovision-xt; RRID: SCR_000441
Statistica10	Statsoft	
Prism 7	Graphpad	
**Other**		
Micro-syringe pump	World Precision Instruments	
LRS-0473-GFM-00050-05 LabSpec 473nm DPSS Laser System Output: >50mW	Laserglow Technologies	R470505GX
LSG-532-NF-7 Fit-Over Safety Goggles 532nmLRS-0473-GFM-00050-05 LabSpec 473nm DPSS Laser System Output: >50mW	Laserglow TechnologiesLaserglow Technologies	AGF5327XXR470505GX
FC/PC Fiber Coupler/CollimatorLSG-532-NF-7 Fit-Over Safety Goggles 532nm	Laserglow TechnologiesLaserglow Technologies	ACFVISHXXAGF5327XX

Optogenetics TTL Pulse Generator - 4 channelsFC/PC Fiber Coupler/Collimator	Doric Lenses Inc.Laserglow Technologies	OTPG_4ACFVISHXX
1x2 Fiber-optic Rotary JointsOptogenetics TTL Pulse Generator - 4 channels	Doric Lenses Inc.Doric Lenses Inc.	FRJ_1x2i_FC-2FC_0.22OTPG_4
Power Meter, Si Sensor, 400 - 1100 nm, 50 nW - 50mW1x2 Fiber-optic Rotary Joints	ThorlabsDoric Lenses Inc.	PM120DFRJ_1x2i_FC-2FC_0.22
0.39 NA, Ø200 µm Core Multimode Optical Fiber, High OH for 300 - 1200 nm, TECS CladPower Meter, Si Sensor, 400 - 1100 nm, 50 nW - 50mW	Thorlabs	FT200UMTPM120D
Ceramic LC MM Ferrule, ID 230µm - 10 pack0.39 NA, Ø200 µm Core Multimode Optical Fiber, High OH for 300 - 1200 nm, TECS Clad	Thorlabs	CFLC230-10FT200UMT
Fiber Inspection Scope 200x, with FC and SMA AdaptersCeramic LC MM Ferrule, ID 230µm - 10 pack	Thorlabs	FS201CFLC230-10
LC Adapter for FS200 Series Fiber Inspection ScopeFiber Inspection Scope 200x, with FC and SMA Adapters	Thorlabs	FS200-LCFS201
White Dust Caps for Ø1.25 mm Ferrules 25packLC Adapter for FS200 Series Fiber Inspection Scope	Thorlabs	CAPLFS200-LC
Fiber Stripping ToolWhite Dust Caps for Ø1.25 mm Ferrules 25pack	Thorlabs	T12S21CAPL
Rubber Gripper for Bare FiberFiber Stripping Tool	Thorlabs	BFG1<br>T12S21
Crimp ToolRubber Gripper for Bare Fiber	Thorlabs	CT042BFG1
Ruby Fiber ScribeCrimp Tool	Thorlabs	S90RCT042
FC/PC and SC/PC Connector Polishing DiscRuby Fiber Scribe	Thorlabs	D50-FCS90R
LC/PC Connector Polishing DiscFC/PC and SC/PC Connector Polishing Disc	Thorlabs	D50-LCD50-FC
6" x 6" Final Lapping (Polishing) Sheets, 0.02 µm	Thorlabs	LFCFD50-LC

Grit (5 Sheets)LC/PC Connector Polishing Disc		
6" x 6" Diamond Lapping (Polishing) Sheets, 1 µm Grit (5 Sheets)6" x 6" Final Lapping (Polishing) Sheets, 0.02 µm Grit (5 Sheets)	Thorlabs	LF1DLFCF
6" x 6" Diamond Lapping (Polishing) Sheets, 3 µm Grit (5 Sheets)6" x 6" Diamond Lapping (Polishing) Sheets, 1 µm Grit (5 Sheets)	Thorlabs	LF3DLF1D
6" x 6" Diamond Lapping (Polishing) Sheets, 6 µm Grit (5 Sheets)6" x 6" Diamond Lapping (Polishing) Sheets, 3 µm Grit (5 Sheets)	Thorlabs	LF6DLF3D
6" x 6" Diamond Lapping (Polishing) Sheets, 30 µm Grit (5 Sheets)6" x 6" Diamond Lapping (Polishing) Sheets, 6 µm Grit (5 Sheets)	Thorlabs	LF30DLF6D
LOCTITE® EPOXY INSTANT MIX™ 5 MINUTE6" x 6" Diamond Lapping (Polishing) Sheets, 30 µm Grit (5 Sheets)	Thorlabs	LF30D
Glass Polishing Plate, 9.5" x 13.5"LOCTITE® EPOXY INSTANT MIX™ 5 MINUTE	Thorlabs	CTG913
Polishing Pad for PC Finishes, 8.75" x 13", 50 DurometerGlass Polishing Plate, 9.5" x 13.5"	Thorlabs	NRS913A<br>CTG913
Crimp ToolPolishing Pad for PC Finishes, 8.75" x 13", 50 Durometer	Thorlabs	CT042NRS913A
FC/PC Multimode Connector, Ø240 µm Bore, Ceramic FerruleCrimp Tool	Thorlabs	30240C1CT042
FC/PC Multimode Connector, Ø240 µm Bore, Ceramic Ferrule	Thorlabs	30240C1

Supplementary References:

1. Gonzalez-Reyes, L.E., et al., *Sonic hedgehog maintains cellular and neurochemical homeostasis in the adult nigrostriatal circuit.* Neuron, 2012. **75**(2): p. 306-19.
