# Supplementary material for: Dopaminergic co-transmission with sonic hedgehog inhibits abnormal involuntary movements in models of Parkinson’s disease and L-Dopa induced dyskinesia
Source: Commun Biol. 2021 Sep 22;4:1071. doi: 10.1038/s42003-021-02567-3 (PMC8458306; doi:10.1038/s42003-021-02567-3)
Supplement: Supplementary file 3 — Description of Additional Supplementary Files [file 42003_2021_2567_MOESM3_ESM.pdf]

Description of Additional Supplementary Files

File name: Supplementary Movie 1

Description: OID AIMs day 1.

File name: Supplementary Movie 2

Description: OID AIMs day 10.

File name: Supplementary Movie 3

Description: OID AIMs after SAG day 11.

File name: Supplementary Data 1.

Description: Source data.
