# Supplementary material for: Dopaminergic co-transmission with sonic hedgehog inhibits abnormal involuntary movements in models of Parkinson’s disease and L-Dopa induced dyskinesia
Source: Commun Biol. 2021 Sep 22;4:1071. doi: 10.1038/s42003-021-02567-3 (PMC8458306; doi:10.1038/s42003-021-02567-3)
Supplement: Supplementary file 7 — Reporting Summary [file 42003_2021_2567_MOESM7_ESM.pdf]

nature research

Corresponding author(s): Andreas H. Kottmann

Last updated by author(s): 08/03/2021

Reporting Summary

Nature Research wishes to improve the reproducibility of the work that we publish. This form provides structure for consistency and transparency in reporting. For further information on Nature Research policies, see Authors & Referees and the Editorial Policy Checklist.

Statistics

For all statistical analyses, confirm that the following items are present in the figure legend, table legend, main text, or Methods section.

n/a	Confirmed	
☐	☒	The exact sample size (*n*) for each experimental group/condition, given as a discrete number and unit of measurement
☐	☒	A statement on whether measurements were taken from distinct samples or whether the same sample was measured repeatedly
☐	☒	The statistical test(s) used AND whether they are one- or two-sided<br>*Only common tests should be described solely by name; describe more complex techniques in the Methods section.*
☐	☒	A description of all covariates tested
☐	☒	A description of any assumptions or corrections, such as tests of normality and adjustment for multiple comparisons
☐	☒	A full description of the statistical parameters including central tendency (e.g. means) or other basic estimates (e.g. regression coefficient) AND variation (e.g. standard deviation) or associated estimates of uncertainty (e.g. confidence intervals)
☐	☒	For null hypothesis testing, the test statistic (e.g. *F*, *t*, *r*) with confidence intervals, effect sizes, degrees of freedom and *P* value noted<br>*Give P values as exact values whenever suitable.*
☒	☐	For Bayesian analysis, information on the choice of priors and Markov chain Monte Carlo settings
☒	☐	For hierarchical and complex designs, identification of the appropriate level for tests and full reporting of outcomes
☒	☐	Estimates of effect sizes (e.g. Cohen's *d*, Pearson's *r*), indicating how they were calculated

Our web collection on statistics for biologists contains articles on many of the points above.

Software and code

Policy information about availability of computer code

Data collection	Zeiss Zen Black, 2015, 2.5<br>Image J, NIH, https://imagej.nih.gov/ij/; RRID: SCR_003070;<br>EthoVison 10 XT, Noldus, http://www.noldus.com/animal-behavior-research/ products/ethovision-xt; RRID: SCR_000441
Data analysis	Prism 8.2.1, Graphpad, https://www.graphpad.com/scientific-software/prism/

For manuscripts utilizing custom algorithms or software that are central to the research but not yet described in published literature, software must be made available to editors/reviewers. We strongly encourage code deposition in a community repository (e.g. GitHub). See the Nature Research guidelines for submitting code & software for further information.

Data

Policy information about availability of data

All manuscripts must include a data availability statement. This statement should provide the following information, where applicable:

- Accession codes, unique identifiers, or web links for publicly available datasets
- A list of figures that have associated raw data
- A description of any restrictions on data availability

All data that support the finding of this study are available upon request from the corresponding author, Andreas H. Kottmann (akottmann@med.cuny.edu).

Field-specific reporting

Please select the one below that is the best fit for your research. If you are not sure, read the appropriate sections before making your selection.

☒ Life sciences ☐ Behavioural & social sciences ☐ Ecological, evolutionary & environmental sciences

For a reference copy of the document with all sections, see nature.com/documents/nr-reporting-summary-flat.pdf

Life sciences study design

All studies must disclose on these points even when the disclosure is negative.

Sample size	We did not use a statistical method to determine sample size a priori. We modeled sample size based on previous publications that uitlized the similar in vivo models for similar analyses.
Data exclusions	In the experiments based on stereotactic neurotoxin injections or stereotactic adenovirus transduction data from fewer than 5 % of animals were excluded due to insufficient neurodegeneration or lack of detectable transgene expression in the targeted brain region upon post mortem verification.
Replication	Data was collected from multiple experiments. No experiments failed to replicate.
Randomization	Animals were randomized across litters and home cages.
Blinding	Behavioral data analyses and cytohistological marker quantifications were conducted blinded to genotype and/or treatment condition

Reporting for specific materials, systems and methods

We require information from authors about some types of materials, experimental systems and methods used in many studies. Here, indicate whether each material, system or method listed is relevant to your study. If you are not sure if a list item applies to your research, read the appropriate section before selecting a response.

Materials & experimental systems

n/a	Involved in the study	
☐	☒	Antibodies
☒	☐	Eukaryotic cell lines
☒	☐	Palaeontology
☐	☒	Animals and other organisms
☒	☐	Human research participants
☒	☐	Clinical data

Methods

n/a	Involved in the study	
☒	☐	ChIP-seq
☒	☐	Flow cytometry
☒	☐	MRI-based neuroimaging

Antibodies

Antibodies used	Rabbit anti-TH, Millipore, #657012;<br>Chicken anti-B Galactosidase, Millipore, # AB986;<br>Goat anti-ChAT, Millipore, AB144P<br>Goat anti-Parv, Swant, # PVG-213;<br>Rabbit anti-p-p44/42 MAPK (Erk1/2), Cell Signaling Technology, # 9101;<br>Rabbit anti-cFosB, Cell Signaling Technology, # 2250;<br>Mouse anti-NeuN, Chemicon international, MAB377
Validation	All antibodies and sera have been extensively validated and utilized for staining the same target tissues analyzed in the current paper in multiple publications before. The results from our use of these reagents is consistent with the published specificity of these reagents.

Animals and other organisms

Policy information about studies involving animals; ARRIVE guidelines recommended for reporting animal research

Laboratory animals	Mouse: WT: C57BL/6J, JAX:000664;<br>Mouse: Shh-nLZL/L; Kottmann Lab<br>Mouse: Dat-Cre Slc6a3tm1(cre)Xz/JM, JAX 020080;<br>Mouse: R26-myrGFP; JAX: 007576<br>Mouse: Shh-GFP-Cre; JAX: 005622<br>Mouse: Pitx3ak/ak; Dr. Un Jung Kang, New York University

	Mouse: SmoL/L, JAX: 004526<br>Mouse: R26SmoM2+/-; JAX: 005130<br>Mouse: ChAT-Ires-Cre, JAX: 006410;<br>Non-human primates: Macaca Fascularis; Xierxin, Beijing, PR of China
Wild animals	*Provide details on animals observed in or captured in the field; report species, sex and age where possible. Describe how animals were caught and transported and what happened to captive animals after the study (if killed, explain why and describe method; if released, say where and when) OR state that the study did not involve wild animals.*
Field-collected samples	*For laboratory work with field-collected samples, describe all relevant parameters such as housing, maintenance, temperature, photoperiod and end-of-experiment protocol OR state that the study did not involve samples collected from the field.*
Ethics oversight	Mouse: Animal use and procedures were in accordance with the National Institutes of Health guidelines and approved by the Institutional Animal Care and Use Committees (IACUC) of the City College of New York / City University of New York.<br>Non-human primates: Macaque experiments were performed in accordance with the European Union directive of September 22, 2010 (2010/63/EU) on the protection of animals used for scientific purposes in an AAALAC-accredited facility following acceptance of study design by the Institute of Lab Animal Science (Chinese Academy of Science, Beijing, China).

Note that full information on the approval of the study protocol must also be provided in the manuscript.
